# Using external morphology as a proxy for stomach size in *Hemigrapsus sanguineus*

**DOI:** 10.1002/ece3.11344

**Published:** 2024-05-02

**Authors:** Laura S. Fletcher, April M. H. Blakeslee, Laura C. Crane, Michele F. Repetto, Benjamin J. Toscano, Blaine D. Griffen

**Affiliations:** ^1^ Department of Biology Brigham Young University Provo Utah USA; ^2^ Department of Biology East Carolina University Greenville North Carolina USA; ^3^ Wells National Estuarine Research Reserve Wells Maine USA; ^4^ Department of Biology Temple University Philadelphia Pennsylvania USA; ^5^ Department of Biology Trinity College Hartford Connecticut USA

**Keywords:** diet quality, gut morphology, *Hemigrapsus sanguineus*, individual variation, progastric carapace region

## Abstract

Stomach morphology can provide insights into an organism's diet. Gut size or length is typically inversely related to diet quality in most taxa, and has been used to assess diet quality in a variety of systems. However, it requires animal sacrifice and time‐consuming dissections. Measures of external morphology associated with diet may be a simpler, more cost‐effective solution. At the species level, external measures of the progastric region of the carapace in brachyuran crabs can predict stomach size and diet quality, with some suggestion that this approach may also work to examine individual diet preferences and specialization at the individual level; if so, the size of the progastric region could be used to predict trends in diet quality and consumption for individuals, which would streamline diet studies in crabs. Here, we tested whether external progastric region size predicts internal stomach size across latitude and time of year for individuals of the invasive Asian shore crab *Hemigrapsus sanguineus*. We found that the width of the progastric region increased at a faster rate with body size than stomach width. In addition, the width of the progastric region followed different trends across sites and over time compared to stomach width. Our results therefore suggest that the progastric region may not be used as a proxy for stomach size variation across individuals.

## Graphical abstract

Diet studies often rely on internal measurements of gut morphology to estimate diet, but this involves time‐consuming dissections and animal sacrifice. The progastric region of the external carapace in brachyuran crabs has been shown to be an effective predictor of diet across species, but not at the individual level. Here, using *Hemigrapsus sanguineus* as a model species, we demonstrate that the progastric region may not be able to predict diet at the individual level.

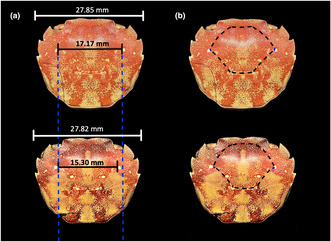

## 1 INTRODUCTION

The quality of an individual's diet determines the amount of energy and nutrients available for processes such as metabolism, growth, and reproduction. In many cases, lower diet quality leads to a reduction in reproductive output, growth, and survival (Cruz‐Rivera & Hay, 2000; Rand et al., 2010). Such consequences for fitness can be short‐ or long‐term; in some marine species, the effects of a poor quality diet negatively impact breeding efforts months down the line, resulting in fewer offspring and offspring with lower survival rates (Sorensen et al., 2009). Diet quality may also influence foraging strategies (Spitz et al., 2012), dictating how much time must be spent foraging and how much food must be consumed to meet daily metabolic demands. General estimates of diet quality therefore provide insight into basic biological processes, such as reproduction and behavior.

Diet quality is not static and can differ considerably between populations (Rand et al., 2010), across individuals (Bolnick et al., 2002; Jack & Wing, 2011), and within individuals through time (Williamson & Steinberg, 2012). For instance, populations of Atka mackerel (*Pleurogrammus monopterygius*) exhibit differential consumption and thus growth rates based on the energetic quality of available prey in the area (Rand et al., 2010), and individual sea urchins (*Holopneustes purpurascens*) switch to more nutritious host plants as they grow bigger (Williamson & Steinberg, 2012). Intraspecific variation in diet quality is especially important to recognize in omnivorous species, in which a single individual may consume prey at multiple trophic levels (e.g., Jack & Wing, 2011). Individual diet quality may also be driven by other factors, including nonlethal injury (Juanes & Hartwick, 1990) and population density that influences the degree of intraspecific competition for resources (Tinker et al., 2008). Given individual diet variation and its impacts on individuals' fitness, studying diet quality at the individual level rather than solely at the species level can reveal the impacts of diet on a species' success (Tinker et al., 2008).

Gut size is frequently used to approximate diet quality. In general, stomach or gut size is expected to vary inversely with diet quality because diets that contain more plant or detrital matter require more intestinal space, either to increase digestion time or to accommodate larger volumes of food due to compensatory feeding (Sibly, 1981). Empirical studies across a broad range of systems support this expectation (mammals: Gross et al., 1985; reptiles: Kohl et al., 2016; birds: Miller, 1975; fish: Hofer, 1988; arthropods: Yang & Joern, 1994). Further, stomach or gut size provides important ecological insights related to diet within each system. For instance, in marine polychaetes, deposit‐feeding species have larger gut volumes than carnivorous species because depository diets are more refractory (i.e., lower in quality) and require longer digestion times (Penry & Jumars, 1990). Individual variation in diet quality within a species follows the same pattern. For example, longer gut lengths in individual monkeyface prickleback (*Cebidichthys violaceus*) are associated with an ontogenetic shift from carnivory in the juvenile stage to herbivory as adults (Montgomery, 1977). Adult gut size is also plastic within individuals of many species, such as the Eurasian perch (*Perca fluviatilis*), in which the gut lengthens in response to decreased diet quality (Olsson et al., 2007). Gut size is therefore a useful tool for approximating diet quality both at species and individual levels.

**FIGURE 1:**
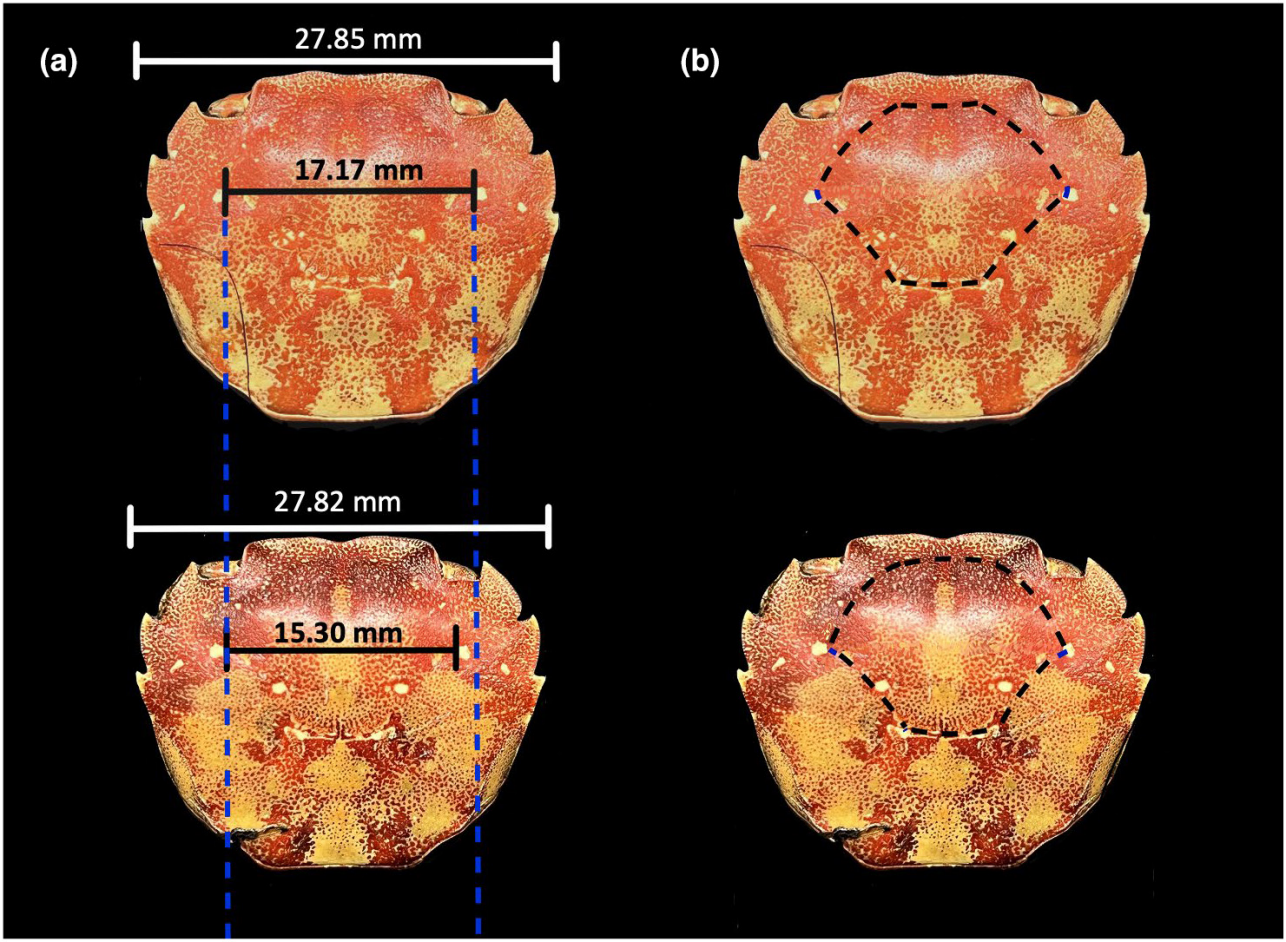
(a) Progastric region widths (in black) and carapace widths (in white) of two examples of *Hemigrapsus sanguineus* individuals used in the study. Vertical blue dotted lines facilitate the comparison between the two external stomach marking widths. (b) The same two crabs pictured in Part (a), but with the progastric region of the carapace outlined in black dotted lines. While the two individuals are nearly identical in carapace width, there is a >10% difference in the width of the progastric carapace region.

Brachyuran crabs are important consumers in many marine systems (Boudreau & Worm, 2012; Howard et al., 2017). As with other animals, stomach size in crabs is negatively correlated with diet quality (Griffen & Mosblack, 2011) and stomach width has therefore been used to successfully assess diet quality for a variety of species (Bas et al., 2014; Cannizzo et al., 2018; Carver et al., 2021; Gül & Griffen, 2020; Reese et al., 2023). However, internal stomach size can only be measured via animal sacrifice. The presence of grooves on the dorsal surface of the carapace in brachyuran crabs is an external morphological trait that represents a potential alternative measure to estimate stomach size (Figure 1), and thus diet quality, and can easily be measured in the field without animal sacrifice. The progastric region of the carapace overlies the cardiac stomach, and unnamed grooves separating the progastric and hepatic carapace regions roughly outline the size and shape of the underlying cardiac stomach (Davie et al., 2015). These grooves likely form during the molting process, as the progastric region is among the first on the new carapace to harden after the molt (Kishori & Reddy, 2003; Knudsen, 1959). The soft carapace may therefore settle around the stomach and, as it hardens, form external grooves that delineate the approximate size and shape of the cardiac stomach. These grooves may reflect internal segmentation, or may reflect sites of muscle attachments, including the gastric muscles (Klompmaker et al., 2019; Patwardhan, 1934; Tshudy & Babcock, 1997). Whatever their origin, these grooves have recently been identified as an accurate predictor of mean diet quality across crab species, with some suggestions that they may also be useful for assessing intraspecific differences in diet quality (Quezada‐Villa et al., 2023). However, the specimens used in that study to assess intraspecific diet did not capture the temporal and spatial variation of individual crab species, which we would expect to influence diet strategy.

Here, we investigate whether the width of the progastric region can be used to assess stomach width as a proxy for diet quality across conspecific individuals, using the invasive Asian shore crab *Hemigrapsus sanguineus* as a model species. This species was first introduced to the coast of New Jersey in 1989, and established populations are now found from Maine (Lord & Williams, 2017) to North Carolina (Epifanio, 2013). One factor contributing to the invasion success of *H. sanguineus* is its broad, generalist diet (Bourdeau & O'Connor, 2003) and ability to switch between high‐ and low‐quality diets (Brousseau & Baglivo, 2005; Griffen et al., 2012, 2015). Griffen and Mosblack (2011) empirically demonstrated that individual of *H. sanguineus* that chose to consume animal material had smaller stomachs than individuals that selected an algal diet. Using this relationship, stomach size has been used to infer changes in diet quality in *H. sanguineus* across season and latitude (Griffen et al., 2012; Reese et al., 2023), as well as across sites that differ in prey availability (Griffen et al., 2020). Thus, it is clear that stomach size can be used to assess diet quality in *H. sanguineus*; however, it is unclear whether size measurements of external morphological traits such as the progastric carapace region could be used to make similar diet predictions.

We tested three separate hypotheses to determine whether the width of the carapace progastric region could predict internal stomach width, a proxy for diet quality: (1) internal stomach width and the width of the progastric region will be positively correlated with each other and with carapace width; (2) internal stomach width and the width of the progastric region will follow qualitatively similar trends over time; and (3) internal stomach width and the width of the progastric region will follow qualitatively similar trends across sites. We tested these hypotheses using 799 adult female crabs collected across the invaded range and across an entire active foraging season. If internal and external metrics exhibit similar trends across space and time, then external morphology may be used as a more efficient and accessible proxy for assessing individual differences in diet quality.

## 2 METHODS

### 2.1 Sampling

We collected 799 adult female crabs from five sites throughout *H. sanguineus*' invasive range: Bailey Island in Harpswell, Maine (43°43′2.7336″ N, 70°0′11.4624″ W; *n* = 157); Odiorne Point State Park in Rye, New Hampshire (43°2′20″ N, 70°42′55″ W; *n* = 157); Goshen Point in Waterford, Connecticut (41°17′56.1″ N, 72°06′44.9″ W; *n* = 288); Cape May Ferry in North Cape May, New Jersey (38°58′3.396″ N, 74°57′45.9858″ W; *n* = 121); and Oregon Inlet, North Carolina (35°46′7.33″ N, 75°31′37.76″ W; *n* = 77). Sampling occurred every other month in March, May, July, September, and November 2020 at all sites but one (Connecticut) to capture the entire reproductive season for the species (Epifanio, 2013; McDermott, 1998). Because the Connecticut site occupies the center of the invaded range, we chose to sample monthly at this site during the same period to capture temporal differences in diet quality at a higher resolution within a single site. Approximately 30 adult females were collected each month of sampling from each site. We identified adults as individuals with a carapace width (CW) greater than 12 mm (McDermott, 1998). We focused on adult females to facilitate other studies for which these same samples were used. Immediately after collection, crabs were placed into individual bags and shipped to Brigham Young University on dry ice, where they were stored at −80°C until dissection. These specimens represent the same crabs previously collected and used by Griffen et al. (2022, 2023) and Reese et al. (2023); however, measurements of the carapace progastric region were collected specifically for use in this study.

### 2.2 Dissections

To prepare for dissections, crabs were thawed in room temperature water. Once thawed, we measured the carapace width (CW) to the nearest 0.01 mm with digital vernier calipers. We then removed the dorsal carapace to access the stomach, which was extracted and placed in an aluminum drying boat. The cardiac stomach width was measured with vernier calipers immediately after removal from the body while the stomach was still wet. We measured stomach width as the widest point along the anterior dorsal margin, following Griffen and Mosblack (2011).

Lastly, after the crabs were fully dried, the width of the progastric region was measured with vernier calipers at its widest point (Figure 1). *H. sanguineus* does not have heavy grooves demarcating this region, as are found in some other species. Rather, the progastric region in *H. sanguineus* is elevated as a bump that rises above the rest of the carapace and is surrounded by light grooves. In individuals where this region was difficult to see with the naked eye, measurements were taken under a dissecting microscope.

### 2.3 Statistical analyses

All analyses were performed in R (v.4.1.2; R Core Team, 2021). For each of the analyses described below, we had to control for the effect of body size on cardiac stomach and progastric region width. Previous diet studies in this species based on cardiac stomach size have used either residual stomach size after accounting for CW, or standardized stomach width (stomach width divided by body width, a unitless ratio) (Griffen et al., 2020; Griffen & Mosblack, 2011; Reese et al., 2023). However, when the standardized approach was used for the progastric region (i.e., progastric width divided by CW), this standardized metric increased with CW. We therefore used residual stomach width and residual progastric region width in all of our analyses, which detrend any relationship between stomach size or progastric region width and body size. A positive residual indicates a larger‐than‐expected stomach or progastric region for a given‐size crab, while a negative residual indicates a smaller stomach or progastric region than anticipated based on body size. We obtained residual stomach size and residual progastric region size for each individual crab from independent regressions of cardiac stomach width and the width of the progastric region against CW.

### 2.4 Relationship between cardiac stomach and progastric widths (Hypothesis 1)

Quezada‐Villa et al. (2023) identified a positive correlation between cardiac stomach width and progastric width in *H. sanguineus*. To determine whether our dataset followed the same pattern, we used a linear model with residual cardiac stomach width as the response variable and residual progastric width as the predictor variable. We also tested whether cardiac stomach width and progastric width increased with CW at similar rates. To do this, we fit a pair of linear models to our data. Both included CW as the predictor variable, with one including cardiac stomach width as the response variable and the other including progastric region width as the response variable. To determine whether any differences between these two models were significant, we followed with an analysis of covariance (ANCOVA), using stomach width as the response variable and carapace width, width type (progastric region or cardiac stomach), and the interaction between the two as predictor variables.

### 2.5 Diet quality over time (Hypothesis 2)

We tested the hypothesis that progastric region width and stomach width would show similar patterns over time. We did this using data from the Connecticut site because of its greater temporal sampling resolution. For this hypothesis and the next, two paired models were fitted to the data: one with cardiac stomach width as the response variable, and the other with progastric region width as the response variable. We then compared the statistical and graphical results of the two paired analyses to determine whether actual stomach size and the width of the progastric region revealed similar qualitative patterns.

We used residual cardiac stomach width and residual progastric region width as response variables in separate linear polynomial models to examine the nonlinear relationship between diet quality and Julian date. We fit first‐, second‐, third‐, and fourth‐order polynomials to both of these response variables and then selected the best‐fitting model for each using the Akaike information criterion (AIC). The highest order polynomial used was determined by visually inspecting the data and was based on the number of distinct changes in slope visible on the graph.

### 2.6 Diet quality across sites (Hypothesis 3)

Using these same crabs, Reese et al. (2023) demonstrated that standardized stomach size differed between sites throughout the invaded range. In our study, we tested whether residual stomach size and residual progastric region width show similar patterns between sites with two separate one‐way ANOVAs followed by Tukey's HSD tests, again using residuals to control for differences in crab size.

## 3 RESULTS

### 3.1 Relationship between body size and the stomach and progastric widths (Hypothesis 1)

**FIGURE 2:**
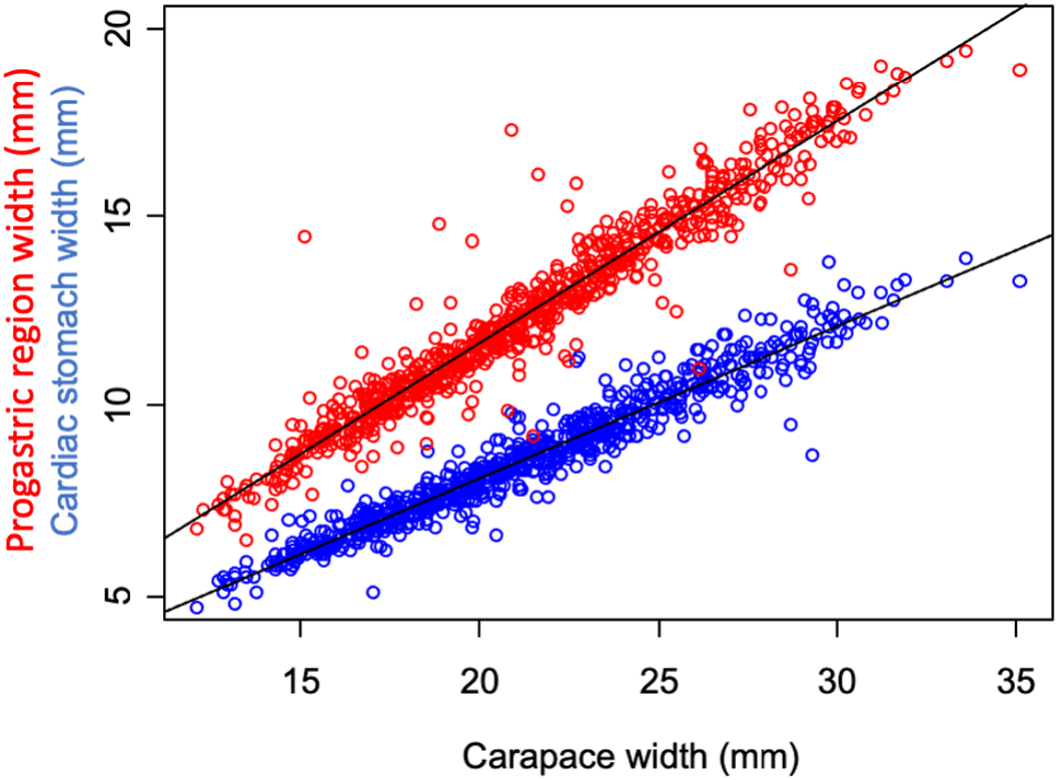
Cardiac stomach width (in blue) and progastric region width (in red) as a function of carapace width. Regression lines are included in black for both cardiac stomach width and external stomach marking width.

Stomach width and the width of the progastric region both increased with body size. However, the width of the progastric region increased faster with body size than stomach width did, as demonstrated by the significant interaction we identified between carapace width and gut metric type (ANCOVA, *t* = 23.760, *p* < .0001). Specifically, cardiac stomach width increased by 0.397 ± 0.004 mm for every mm increase in carapace width (*t* = 94.512, *p* < .0001, Figure 2), while the width of the progastric region increased by 0.583 ± 0.006 mm for every mm increase in carapace width (*t* = 100.845, *p* < .0001, Figure 2).

**FIGURE 3:**
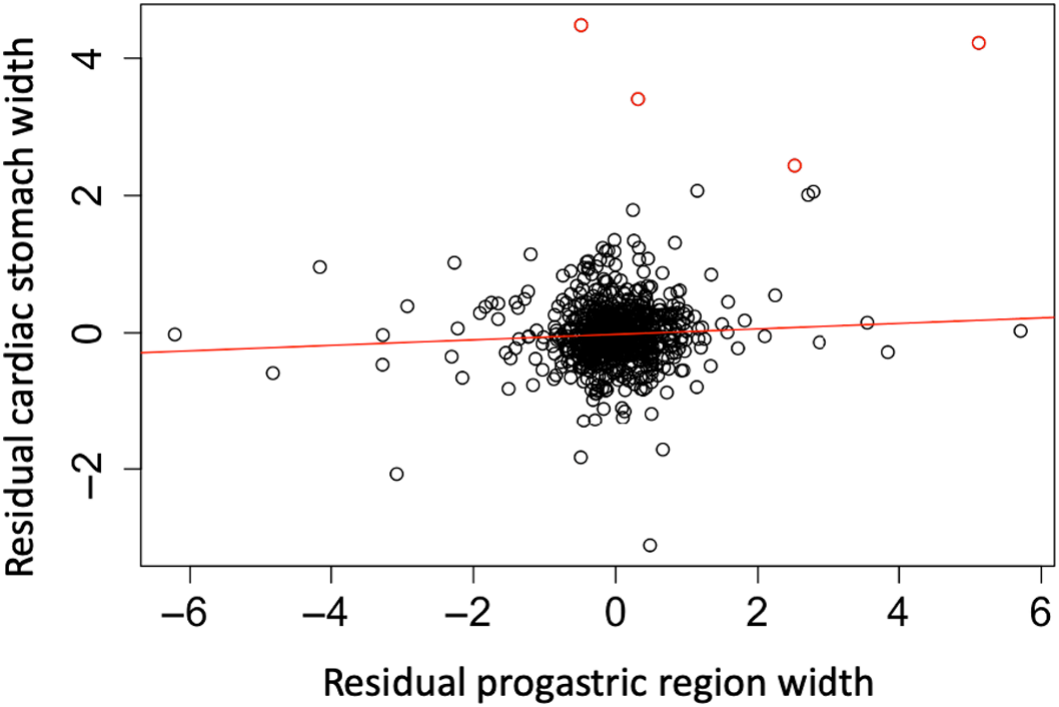
Residual cardiac stomach width as a function of residual progastric region width. A regression line is included in red. Outliers excluded from the final analysis have been highlighted in red.

After accounting for carapace width, we identified no significant relationship between residual stomach width and progastric region width (*t* = 1.831, *p* = .0676; Figure 3). Four outliers had strong influences on the results, so were removed from this and all subsequent analyses (red points in Figure 3).

### 3.2 Diet quality over time (Hypothesis 2)

**FIGURE 4:**
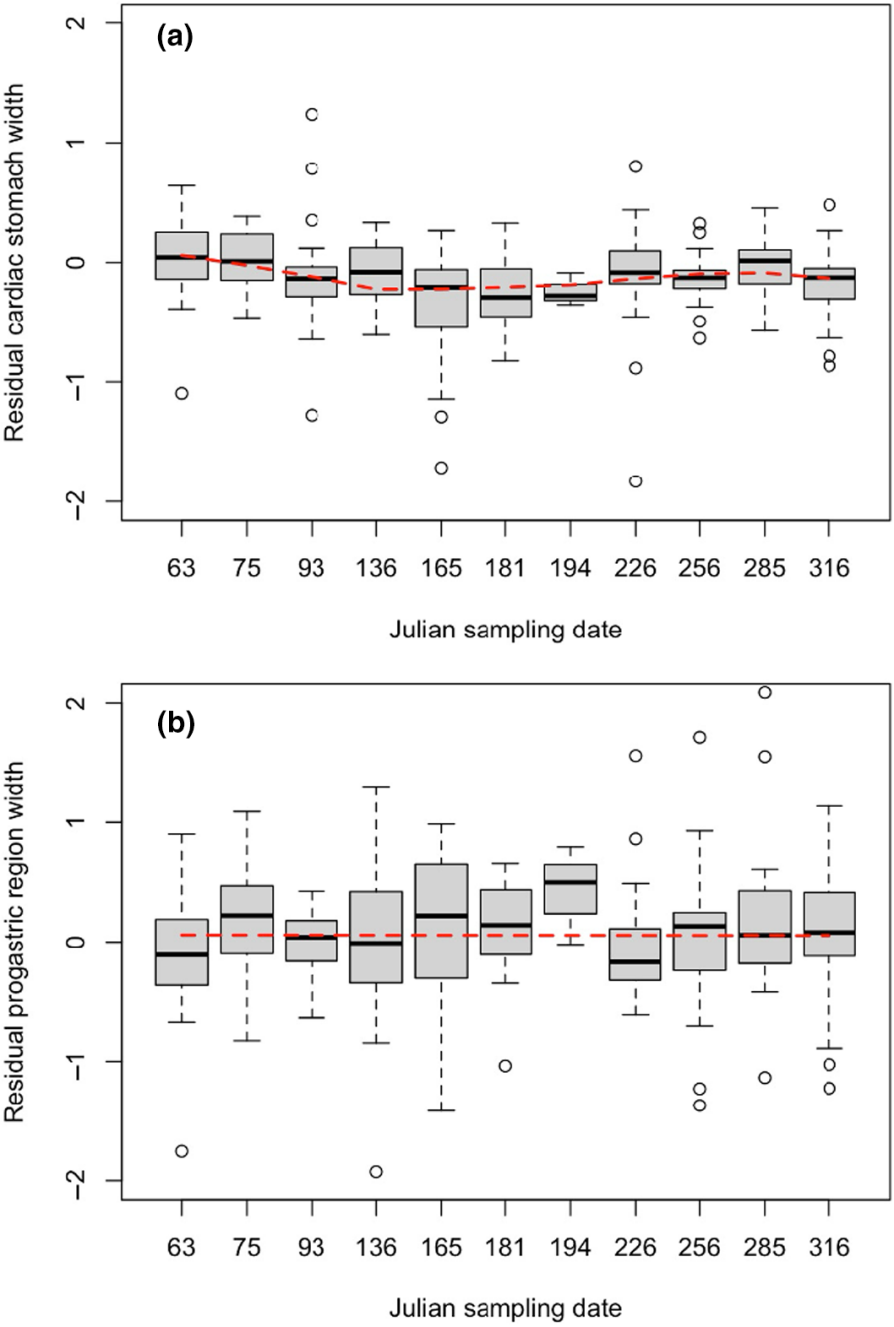
Residual cardiac stomach width (a) and residual progastric region width (b) as a function of sampling date in *Hemigrapsus sanguineus* for individuals collected from Goshen Point in Waterford, Connecticut. Boxes represent the interquartile range, whiskers represent 1.5 × the interquartile range, open circles denote outliers, and black bars represent medians. The dashed red lines are polynomial regression curves fitted to the data.

While both stomach width and the width of the progastric region varied through time with the sampling date at the Connecticut site (Figure 4), the trends between these two metrics differed considerably. The best‐fitting polynomial regression model for residual stomach width included the first‐ (*t* = −3.159, *p* = .0018), second‐ (*t* = 2.803, *p* = .0054), and third‐order (*t* = −2.501, *p* = .0129) polynomial terms of Julian date as predictor variables (Figure 4a), suggesting that stomach width, and by extension, diet quality, varies seasonally with two periods of decrease and one short period of increase throughout the year (Figure 4). The third‐ and fourth‐order polynomials were not statistically different (ΔAIC 1.605), but the fourth‐order term was not significant (*t* = −1.888, *p* = .060). Thus, we chose (based on Occam's Razor) the simpler model with three polynomial terms. The ΔAIC of the next best model (which included only the first‐ and second‐order polynomial terms) was 4.277.

In contrast, there was more variation in the progastric measurements, and consequently, the best‐fitting model included only the first‐order polynomial term of Julian date as a predictor variable, which was not significant (*t* = − 0.080; *p* = .937, Figure 4b). The width of the progastric region, therefore, did not vary significantly over time. Again, the second‐order model was not distinguishable from the first‐order model (ΔAIC 1.976), and both terms in this model were not significant (*p* > .8); we therefore accepted the simpler model. The ΔAIC of the next best model (third order polynomial) was 3.398.

### 3.3 Diet quality across sites (Hypothesis 3)

**FIGURE 5:**
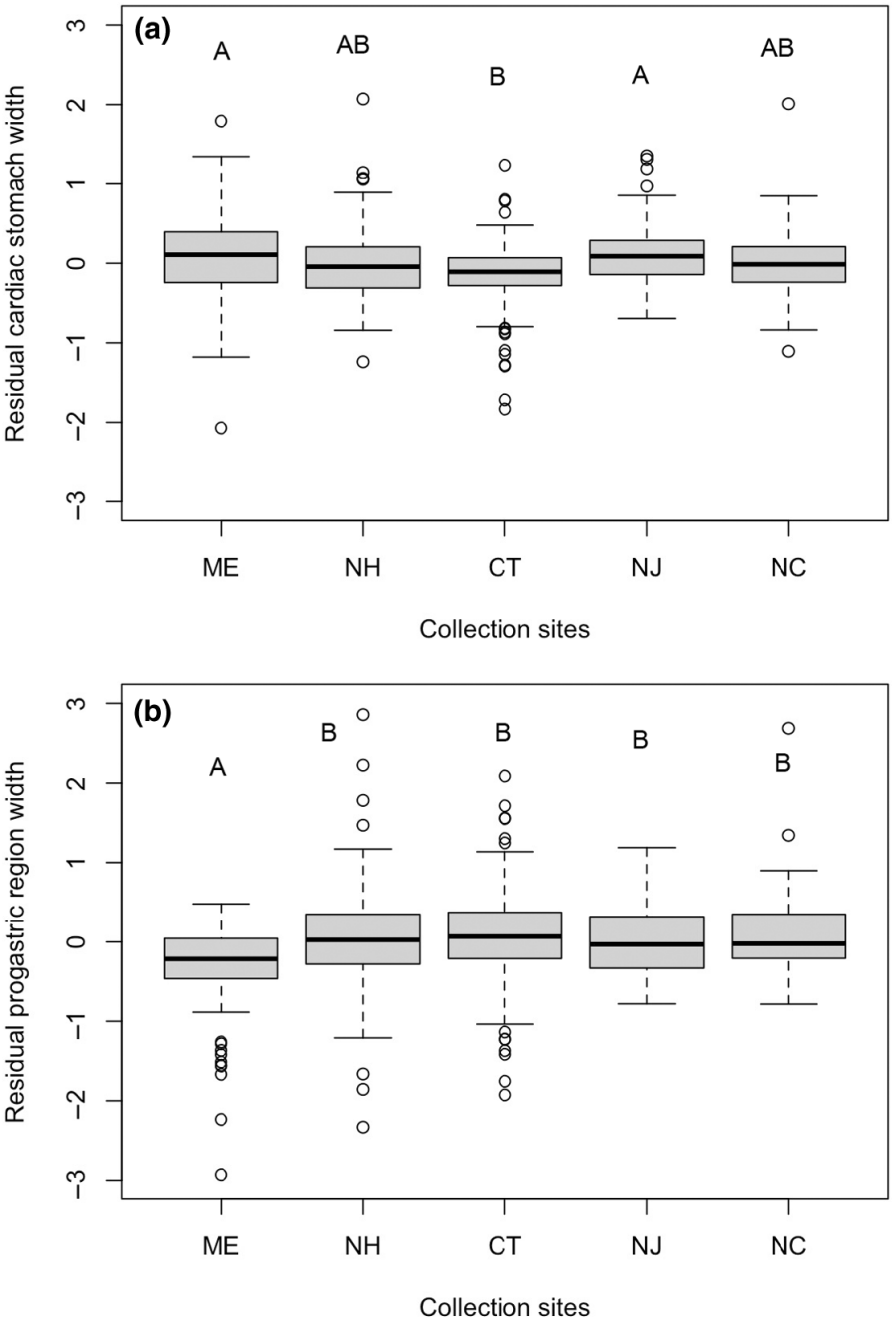
Residual cardiac stomach width (a) and residual progastric region width (b) by location in *Hemigrapsus sanguineus*. Upper case letters denote sites at which residual external stomach marking width or residual stomach width did not differ significantly based on Tukey's HSD tests for each metric independently (*p* < .05). Outliers falling outside the maximum and minimum *y*‐axis values were excluded from the graph to enhance trends. Boxplots are as explained in the Figure 3 caption.

Both stomach width (ANOVA *F*_4,778_ = 7.233, *p* < .0001) and width of the progastric carapace region (ANOVA *F*_4,778_ = 6.272, *p* < .0001) differed across collection sites, though these differences were not the same (Figure 5). Mean stomach width was significantly larger in Maine and New Jersey than in Connecticut (*p* < .0001 and *p* = .0010, respectively; Figure 5), while mean progastric width was significantly smaller in Maine compared to New Hampshire, Connecticut, North Carolina, and New Jersey (*p* = .0009, *p* < .0001, *p* = .0022, and *p* = .0099, respectively; Figure 5).

## 4 DISCUSSION

Using external morphology as a substitute for internal stomach morphology in ecological diet studies may reduce collection time, effort, and animal sacrifice. The width of the progastric region of the carapace is an effective predictor of stomach width at the species level (Quezada‐Villa et al., 2023), but its relationship with stomach width at the individual level and across time and space has not been tested until now. We have shown here that both stomach width and the width of the progastric region increased with body size, though progastric width increased at a greater rate (Hypothesis 1). However, contrary to our hypothesis and to the findings of Quezada‐Villa et al. (2023), we found no significant relationship between residual cardiac stomach width and residual progastric region width (Hypothesis 1). We also found that the two metrics exhibited different trends over time and across sites (Hypothesis 2 and 3).

Our results may differ from those of Quezada‐Villa et al. (2023) due to our larger sample size (*n* = 799) and geographic breadth. The lack of correlation between internal and external morphology and the more rapid increase in progastric region width with body size compared to the cardiac stomach (Hypothesis 1) may be explained by the fact that crabs that consume higher quality diets have smaller stomachs (Griffen & Mosblack, 2011). At the same time, a higher quality diet should lead to higher growth rates (Catacutan, 2002), causing the crab's body to grow faster (and the progastric region along with it), while its stomach remains relatively small in comparison. Further light may be shed on this relationship by investigating the formation of the progastric region and how it changes across molts for crabs that eat different diets, though this was outside the scope of this study.

The relationship between the progastric carapace region and diet may also be influenced by external factors. The intermolt period and relationship between external and internal morphology in crabs may vary across sites for several reasons, including differences in temperature (Kuhn & Darnell, 2019). The intermolt period in other species of crabs, such as *Callinectes similis*, is known to decrease at higher temperatures (Kuhn & Darnell, 2019). Assuming these patterns also hold for *H. sanguineus*, it is therefore possible that at the southern end of the range, where average temperatures are higher and intermolt periods may be shorter, the size of the progastric region may be more closely related to stomach size. Another factor that could influence the intermolt period and the relationship between external and internal gut morphology is food availability. Food availability affects growth in this species (Griffen et al., 2020), which may in turn alter the length of the intermolt period. Sites at the northern edge of the invasive range with higher food availability and growth rates may have shorter intermolt periods, and more closely related internal and external gut morphologies. However, this suggests that both northern and southern sites should have shorter intermolt periods and that we should expect a close relationship between internal and external morphology throughout the invasive range. Given that this is not what we see here, it is possible that temperature and food availability do not have equal impacts on the intermolt period. One or the other may affect molting intervals more strongly, leading to the disconnect between internal and external morphology we observed across sites. It is also possible that another factor we have not yet identified is interacting with temperature and food availability to produce the observed pattern. Regardless, even if variation in temperature and food availability across latitudes contributes to the differences we observed across sites (Hypothesis 3), it still does not explain why we observed different trends over time at the same site (Hypothesis 2).

It should be noted that the relationship between external and internal stomach morphology varies across crab species (Quezada‐Villa et al., 2023), so the lack of similarity observed here in these two metrics may not apply to other species. Nevertheless, the goal of this study was to determine whether external morphological measurements could be used as a proxy for stomach size in brachyuran crabs, which itself is used as a proxy for diet strategy across a broad range of organisms. We demonstrate that external morphology and internal stomach morphology are not correlated in *H. sanguineus* after removing the confounding impacts of body size. Further, while both increase linearly with body size, the width of the progastric region increases faster than stomach width. Both metrics vary over space and/or time where diet is expected to be the driving force, though the trends differ. The relationship between stomach size and the progastric region requires further insight before the reasons for the differing trends are understood. However, based on our current understanding, this metric may not be a useful proxy for individual diet variation in brachyuran crabs.

## AUTHOR CONTRIBUTIONS

**Laura S. Fletcher:** Formal analysis (equal); investigation (equal); writing – original draft (lead); writing – review and editing (equal). **April M. H. Blakeslee:** Data curation (equal); investigation (equal); writing – review and editing (equal). **Laura C. Crane:** Data curation (equal); investigation (equal); writing – review and editing (equal). **Michele F. Repetto:** Data curation (equal); investigation (equal); writing – review and editing (equal). **Benjamin J. Toscano:** Data curation (equal); investigation (equal); writing – review and editing (equal). **Blaine D. Griffen:** Conceptualization (lead); data curation (supporting); formal analysis (equal); methodology (lead); project administration (lead); writing – review and editing (equal).

## CONFLICT OF INTEREST STATEMENT

None declared.

## Data Availability

All data used in this manuscript are available on Dryad Digital Repository at https://doi.org/10.5061/dryad.6m905qg5h.

## References

[ece311344-bib-0001] Bas, C. , Lancia, J. P. , Luppi, T. , Méndez‐Casariego, A. , Kittlein, M. , & Spivak, E. (2014). Influence of tidal regime, diurnal phase, habitat and season on feeding of an intertidal crab. Marine Ecology, 35(3), 319–331.

[ece311344-bib-0002] Bolnick, D. I. , Yang, L. H. , Fordyce, J. A. , Davis, J. M. , & Svanbäck, R. (2002). Measuring individual‐level resource specialization. Ecology, 83(10), 2936–2941.

[ece311344-bib-0003] Boudreau, S. A. , & Worm, B. (2012). Ecological role of large benthic decapods in marine ecosystems: A review. Marine Ecology Progress Series, 469, 195–213.

[ece311344-bib-0004] Bourdeau, P. E. , & O'Connor, N. J. (2003). Predation by the nonindigenous Asian shore crab *Hemigrapsus sanguineus* on macroalgae and molluscs. Northeastern Naturalist, 10(3), 319–334.

[ece311344-bib-0005] Brousseau, D. J. , & Baglivo, J. A. (2005). Laboratory investigations of food selection by the Asian shore crab, *Hemigrapsus sanguineus*: Algal versus animal preference. Journal of Crustacean Biology, 25(1), 130–134.

[ece311344-bib-0006] Cannizzo, Z. J. , Dixon, S. R. , & Griffen, B. D. (2018). An anthropogenic habitat within a suboptimal colonized ecosystem provides improved conditions for a range‐shifting species. Ecology and Evolution, 8(3), 1521–1533. 29435229 10.1002/ece3.3739 PMC5792588

[ece311344-bib-0007] Carver, J. , Meidell, M. , Cannizzo, Z. J. , & Griffen, B. D. (2021). Evidence for use of both capital and income breeding strategies in the mangrove tree crab, *Aratus pisonii* . Scientific Reports, 11(1), 14576. 34272445 10.1038/s41598-021-94008-8 PMC8285475

[ece311344-bib-0008] Catacutan, M. R. (2002). Growth and body composition of juvenile mud crab, *Scylla serrata*, fed different dietary protein and lipid levels and protein to energy ratios. Aquaculture, 208(1–2), 113–123.

[ece311344-bib-0009] Cruz‐Rivera, E. , & Hay, M. E. (2000). Can quantity replace quality? Food choice, compensatory feeding, and fitness of marine mesograzers. Ecology, 81(1), 201–219.

[ece311344-bib-0010] Davie, P. J. , Guinot, D. , & Ng, P. K. (2015). Anatomy and functional morphology of Brachyura. In P. Castro , P. J. F. Davie , D. Guinot , F. Schram , & C. Von Vaupel Klein (Eds.), Treatise on zoology – Anatomy, taxonomy, biology. The Crustacea, volume 9 part C (2 vols, pp. 11–163). Brill.

[ece311344-bib-0011] Epifanio, C. E. (2013). Invasion biology of the Asian shore crab *Hemigrapsus sanguineus*: A review. Journal of Experimental Marine Biology and Ecology, 441, 33–49.

[ece311344-bib-0012] Griffen, B. D. , Alder, J. , Anderson, L., III , Asay, E. G. , Blakeslee, A. , Bolander, M. , Cabrera, D. , Carver, J. , Crane, L. C. , DiNuzzo, E. R. , Fletcher, L. S. , Luckett, J. , Meidell, M. , Pinkston, E. , Reese, T. C. , Repetto, M. F. , Smith, N. , Stancil, C. , Tepolt, C. K. , … Vernier, A. (2022). Latitudinal and temporal variation in injury and its impacts in the invasive Asian shore crab *Hemigrapsus sanguineus* . Scientific Reports, 12(1), 16557. 36192531 10.1038/s41598-022-21119-1 PMC9530151

[ece311344-bib-0013] Griffen, B. D. , Altman, I. , Bess, B. M. , Hurley, J. , & Penfield, A. (2012). The role of foraging in the success of invasive Asian shore crabs in New England. Biological Invasions, 14, 2545–2558.

[ece311344-bib-0014] Griffen, B. D. , Bailey, J. , Carver, J. , Vernier, A. , DiNuzzo, E. R. , Anderson, L., III , Meidell, M. , & Potter, B. (2020). Mechanisms of possible self‐limitation in the invasive Asian shore crab *Hemigrapsus sanguineus* . Scientific Reports, 10(1), 16908. 33037256 10.1038/s41598-020-74053-5 PMC7547685

[ece311344-bib-0015] Griffen, B. D. , Bolander, M. , Blakeslee, A. , Crane, L. C. , Repetto, M. F. , Tepolt, C. K. , & Toscano, B. J. (2023). Past energy allocation overwhelms current energy stresses in determining energy allocation trade‐offs. Ecology and Evolution, 13(8), e10402. 37560183 10.1002/ece3.10402 PMC10408252

[ece311344-bib-0016] Griffen, B. D. , & Mosblack, H. (2011). Predicting diet and consumption rate differences between and within species using gut ecomorphology. Journal of Animal Ecology, 80(4), 854–863. 21418211 10.1111/j.1365-2656.2011.01832.x

[ece311344-bib-0017] Griffen, B. D. , Vogel, M. , Goulding, L. , & Hartman, R. (2015). Energetic effects of diet choice by invasive Asian shore crabs: Implications for persistence when prey are scarce. Marine Ecology Progress Series, 522, 181–192.

[ece311344-bib-0018] Gross, J. E. , Wang, Z. , & Wunder, B. A. (1985). Effects of food quality and energy needs: Changes in gut morphology and capacity of *Microtus ochrogaster* . Journal of Mammalogy, 66(4), 661–667.

[ece311344-bib-0019] Gül, M. R. , & Griffen, B. D. (2020). Diet, energy storage, and reproductive condition in a bioindicator species across beaches with different levels of human disturbance. Ecological Indicators, 117, 106636.

[ece311344-bib-0020] Hofer, R. (1988). Morphological adaptations of the digestive tract of tropical cyprinids and cichlids to diet. Journal of Fish Biology, 33(3), 399–408.

[ece311344-bib-0021] Howard, B. R. , Therriault, T. W. , & Côté, I. M. (2017). Contrasting ecological impacts of native and non‐native marine crabs: A global meta‐analysis. Marine Ecology Progress Series, 577, 93–103.

[ece311344-bib-0022] Jack, L. , & Wing, S. R. (2011). Individual variability in trophic position and diet of a marine omnivore is linked to kelp bed habitat. Marine Ecology Progress Series, 443, 129–139.

[ece311344-bib-0023] Juanes, F. , & Hartwick, E. B. (1990). Prey size selection in Dungeness crabs: The effect of claw damage. Ecology, 71(2), 744–758.

[ece311344-bib-0024] Kishori, B. , & Reddy, P. S. (2003). Influence of leucine‐enkephalin on moulting and vitellogenesis in the freshwater crab, *Oziotelphusa senex senex* (Fabricius, 1791) (Decapoda, Brachyura). Crustaceana, 76, 1281–1290.

[ece311344-bib-0025] Klompmaker, A. A. , Hyžný, M. , Portell, R. W. , Jauvion, C. , Charbonnier, S. , Fussell, S. S. , Klier, A. T. , Tejera, R. , & Jakobsen, S. L. (2019). Muscles and muscle scars in fossil malacostracan crustaceans. Earth‐Science Reviews, 194, 306–326.

[ece311344-bib-0026] Knudsen, J. W. (1959). Shell formation and growth of the California xanthid crabs. Ecology, 40(1), 113–115.

[ece311344-bib-0027] Kohl, K. D. , Brun, A. , Magallanes, M. , Brinkerhoff, J. , Laspiur, A. , Acosta, J. C. , Bordenstein, S. R. , & Caviedes‐Vidal, E. (2016). Physiological and microbial adjustments to diet quality permit facultative herbivory in an omnivorous lizard. Journal of Experimental Biology, 219(12), 1903–1912. 27307545 10.1242/jeb.138370

[ece311344-bib-0028] Kuhn, A. A. , & Darnell, M. Z. (2019). Elevated temperature induces a decrease in intermolt period and growth per molt in the lesser blue crab *Callinectes similis* Williams, 1966 (Decapoda: Brachyura: Portunidae). Journal of Crustacean Biology, 39(1), 22–27.

[ece311344-bib-0029] Lord, J. P. , & Williams, L. M. (2017). Increase in density of genetically diverse invasive Asian shore crab (*Hemigrapsus sanguineus*) populations in the Gulf of Maine. Biological Invasions, 19, 1153–1168. 28919836 10.1007/s10530-016-1304-1 PMC5597051

[ece311344-bib-0030] McDermott, J. J. (1998). The western Pacific brachyuran *Hemigrapsus sanguineus* (Grapsidae) in its new habitat along the Atlantic coast of the United States: Reproduction. Journal of Crustacean Biology, 18(2), 308–316.

[ece311344-bib-0031] Miller, M. R. (1975). Gut morphology of mallards in relation to diet quality. The Journal of Wildlife Management, 39, 168–173.

[ece311344-bib-0032] Montgomery, W. L. (1977). Diet and gut morphology in fishes, with special reference to the monkeyface prickleback, *Cebidichthys violaceus* (Stichaeidae: Blennioidei). Copeia, 1977(1), 178–182.

[ece311344-bib-0033] Olsson, J. , Quevedo, M. , Colson, C. , & Svanbäck, R. (2007). Gut length plasticity in perch: Into the bowels of resource polymorphisms. Biological Journal of the Linnean Society, 90(3), 517–523.

[ece311344-bib-0034] Patwardhan, S. S. (1934). On the structure and mechanism of the gastric mill in decapoda: I. The structure of the gastric mill in *Paratelphusa guerini* (M. Edw.). Proceedings of the Indian Academy of Sciences, 1(5), 183–196.

[ece311344-bib-0035] Penry, D. L. , & Jumars, P. A. (1990). Gut architecture, digestive constraints and feeding ecology of deposit‐feeding and carnivorous polychaetes. Oecologia, 82, 1–11. 28313130 10.1007/BF00318526

[ece311344-bib-0036] Quezada‐Villa, K. , Cannizzo, Z. J. , Carver, J. , Dunn, R. P. , Fletcher, L. S. , Kimball, M. E. , McMullin, A. L. , Orocu, B. , Pfirrmann, B. W. , Pinkston, E. , Reese, T. C. , Smith, N. , Stancil, C. , Toscano, B. J. , & Griffen, B. D. (2023). Predicting diet in brachyuran crabs using external morphology. PeerJ, 11, e15224. 37065690 10.7717/peerj.15224 PMC10100828

[ece311344-bib-0037] Rand, K. M. , Beauchamp, D. A. , & Lowe, S. A. (2010). Longitudinal growth differences and the influence of diet quality on Atka mackerel of the Aleutian Islands, Alaska: Using a bioenergetics model to explore underlying mechanisms. Marine and Coastal Fisheries, 2(1), 362–374.

[ece311344-bib-0600] R Core Team (2021). R: A language and enviornment for statistical computing. R Foundation for Statistical Computing, Vienna, Austria. https://www.R‐project.org/.

[ece311344-bib-0038] Reese, T. C. , Alder, J. , Asay, E. G. , Blakeslee, A. M. , Cabrera, D. , Crane, L. C. , Fletcher, L. S. , Pinkston, E. , Repetto, M. F. , Smith, N. , Stancil, C. , Tepolt, C. K. , Toscano, B. J. , & Griffen, B. D. (2023). Effects of season and latitude on the diet quality of the invasive Asian shore crab *Hemigrapsus sanguineus* . Marine Ecology Progress Series, 704, 67–79.

[ece311344-bib-0039] Sibly, R. M. (1981). Strategies of digestion and defecation. Physiological ecology (Vol. 169, pp. 109–139). Blackwell Scientific Publications.

[ece311344-bib-0040] Sorensen, M. C. , Hipfner, J. M. , Kyser, T. K. , & Norris, D. R. (2009). Carry‐over effects in a Pacific seabird: Stable isotope evidence that pre‐breeding diet quality influences reproductive success. Journal of Animal Ecology, 78(2), 460–467. 19021778 10.1111/j.1365-2656.2008.01492.x

[ece311344-bib-0041] Spitz, J. , Trites, A. W. , Becquet, V. , Brind'Amour, A. , Cherel, Y. , Galois, R. , & Ridoux, V. (2012). Cost of living dictates what whales, dolphins and porpoises eat: The importance of prey quality on predator foraging strategies. PLoS One, 7(11), e50096. 23185542 10.1371/journal.pone.0050096 PMC3503768

[ece311344-bib-0042] Tinker, M. T. , Bentall, G. , & Estes, J. A. (2008). Food limitation leads to behavioral diversification and dietary specialization in sea otters. Proceedings of the National Academy of Sciences of the United States of America, 105(2), 560–565. 18195370 10.1073/pnas.0709263105 PMC2206575

[ece311344-bib-0043] Tshudy, D. , & Babcock, L. E. (1997). Morphology‐based phylogenetic analysis of the clawed lobsters (family Nephropidae and the new family Chilenophoberidae). Journal of Crustacean Biology, 17(2), 253–263.

[ece311344-bib-0044] Williamson, J. E. , & Steinberg, P. D. (2012). Fitness benefits of size‐dependent diet switching in a marine herbivore. Marine Biology, 159, 1001–1010.

[ece311344-bib-0045] Yang, Y. , & Joern, A. (1994). Gut size changes in relation to variable food quality and body size in grasshoppers. Functional Ecology, 8, 36–45.

